# Structural and Functional Insights into the Biofilm-Associated BceF Tyrosine Kinase Domain from *Burkholderia cepacia*

**DOI:** 10.3390/biom11081196

**Published:** 2021-08-12

**Authors:** Michal Mayer, Yulia Matiuhin, Mickal Nawatha, Orly Tabachnikov, Inbar Fish, Nili Schutz, Hay Dvir, Meytal Landau

**Affiliations:** 1 Department of Biology, Technion-Israel Institute of Technology, Haifa 3200003, Israel; michalmmayer@gmail.com (M.M.); ulikma@gmail.com (Y.M.); mickal.soso@gmail.com (M.N.); orlyt@tx.technion.ac.il (O.T.); 2 Department of Biomolecular Sciences, Weizmann Institute of Science, Rehovot 7610001, Israel; 3 Entoprotech Ltd., 2 Ner Halayla, Caesarea 3088900, Israel; 4 Department of Biochemistry and Molecular Biology, George S. Wise Faculty of Life Sciences, Tel Aviv University, Tel Aviv 6997801, Israel; inbarkaplan@gmail.com; 5 Department of Pharmaceutical Chemistry, University of California San Francisco, 1700 4th Street, San Francisco, CA 94143-2550, USA; 6 Kamari Pharma, Nes Ziona 7403626, Israel; nilischutz@gmail.com; 7 Institute of Animal Science, Volcani Institute, Agricultural Research Organization, Rishon LeZiyon 7528809, Israel; 8 European Molecular Biology Laboratory (EMBL), 22607 Hamburg, Germany

**Keywords:** bacterial tyrosine kinases, *Burkholderia cepacian*, X-ray crystallography, antivirulence

## Abstract

BceF is a bacterial tyrosine kinase (BY-kinase) from *Burkholderia cepacia*, a Gram-negative bacterium accountable for respiratory infections in immunocompromised and cystic fibrosis patients. BceF is involved in the production of exopolysaccharides secreted to the biofilm matrix and promotes resistant and aggressive infections. BY-kinases share no homology with mammalian kinases, and thereby offer a means to develop novel and specific antivirulence drugs. Here, we report the crystal structure of the BceF kinase domain at 1.85 Å resolution. The isolated BceF kinase domain is assembled as a dimer in solution and crystallized as a dimer in the asymmetric unit with endogenous adenosine-diphosphate bound at the active sites. The low enzymatic efficiency measured in solution may be explained by the partial obstruction of the active sites at the crystallographic dimer interface. This study provides insights into self-assembly and the specific activity of isolated catalytic domains. Several unique variations around the active site compared to other BY-kinases may allow for structure-based design of specific inhibitors to target *Burkholderia cepacia* virulence.

## 1. Introduction

*Burkholderia cepacia* (*B. cepacia*) is a group of opportunistic Gram-negative bacteria involved in infections of immunocompromised and cystic fibrosis patients [1,2]. *B. cepacia* can form biofilms, namely bacterial communities attached to surfaces, incorporated by a self-produced extracellular matrix. The biofilm protects the bacteria from disinfectants and host defenses, and thereby increases their resistance to antibiotics [2]. Over the last few decades, the resistance of pathogenic bacteria to antibiotics has expanded and raised serious public health concerns [3]. The reason likely lies in the nature of most antibiotics, which target essential bacterial proteins and activities, thus exerting strong evolutionary pressure for the development of resistant strains [4]. Accordingly, in the battle against bacterial resistance, there is a constant need for the development of novel antibacterial drugs with new modes of action. Targeting key virulent determinants, rather than essential bacterial factors, may potentially reduce the aggressiveness of the infection and exert lower evolutionary pressure for resistance to develop. Another important consideration in the search for novel drugs is targeting bacterial proteins with minimal homology to human proteins in order to increase drug specificity and reduce side effects.

Bacterial tyrosine kinases (BY-kinases), which are present both in Gram-positive and Gram-negative species, meet the above-mentioned criteria for the development of novel antibacterial drugs [3]. BY-kinases control a wide spectrum of metabolic pathways and regulatory mechanisms, such as the metabolism of polysaccharides, DNA metabolism, stress response, and virulence [3,5]. Of utmost interest for antibiotic development is the role of BY kinases as key regulators of biofilm formation and virulence [2,3]. Moreover, since the molecular structure of BY-kinases comprises specific motifs and folds that are not found in eukaryotic kinases, specific inhibition of these enzymes may not significantly affect the host proteins [3,6]. In addition to BY-kinases, which constitute the majority of prokaryotic protein tyrosine kinases, there are two other protein-tyrosine kinase families in bacteria: the Hanks-type, characterized by Hanks motifs, similar to eukaryotic tyrosine kinases [7], and an umbrella family of “odd” protein tyrosine kinases [8].

The cluster of genes named *bce* in *B. cepacia* is essential for exopolysaccharide biosynthesis [2]. The *bceF* gene encodes for the protein tyrosine kinase BceF, which is required for the biosynthesis of the exopolysaccharide cepacian, and accordingly, a *B. cepacia bceF* mutant strain showed reduced biofilm formation [2]. Similar to other BY-kinases in Gram-negative bacteria, BceF is composed of a short cytoplasmic N-terminus, followed by a transmembrane helix, a periplasmic/extracellular domain, a second transmembrane helix, and a cytoplasmic C-terminal catalytic domain [3,6]. In proteobacteria, these regions/domains are expressed as a single polypeptide, whereas in firmicutes they exist as two separate proteins, encoded by the same operon [3,6] (Figure S1). The catalytic cytoplasmic domain contains the Walker A, Walker A’ and Walker B motifs that are also found in ATPases of the MinD/Mrp superfamily [6,9]. Another important region of BY-kinases is located at the C-terminal end of the kinase domain and contains several tyrosine residues that constitute the autophosphorylation sites of BY-kinases, called the Y-cluster [5,10]. The crystal structures of BY-kinase domains of Wzc (PDB code 3LA6 [11]) and Etk (PDB code 3CIO [12]) from E.coli, and the chimeric CapA/B (PDB codes 4JMP and 4JLV [13], and 3BFV and 2VED [14]) from *S. aureus*, revealed a three-dimensional fold that distinguishes them from human kinases. Moreover, it was suggested that BY-kinases function via the formation of a homo-octameric quaternary structure. The current mechanistic model features a cyclic process of inter-molecular trans-auto-phosphorylation within the octamer in which one kinase phosphorylates the C-terminal tyrosine cluster of an adjacent monomer, leading to partial disassociation of the octamer, whereas dephosphorylation via tyrosine phosphatases leads to re-association of the octamer [14]. The conformational changes induced by the reversible phosphorylation, namely expansion and contraction of the cytoplasmic region of the octamer, putatively affect the transmembrane and the periplasmic/extracellular domains, thereby regulating the polymerization and the export of polysaccharides [14].

To investigate the structural and mechanistic features of BceF, we have determined the crystal structure of the recombinant kinase domain of BceF and characterized its enzymatic properties. The reported novel structure, together with our biochemical and bioinformatics analyses, shines new light on the self-assembly of isolated BY-kinase domains, and provides a structural framework to guide the design of inhibitors to target BY-kinases and their role in biofilm formation.

## 2. Materials and Methods

### 2.1. BceF Cloning

The sequence encoding the kinase domain of BceF (UniProt accession number Q0GYW2; residues 471–741) was optimized for expression in *E. coli*. Sequence encoding for the NcoI restriction site, His6-tag, and TEV cleavage site was added at the N-terminus, and BamHI restriction sites were added at the C-terminus of the gene. The synthetic gene was purchased from HyLabs and cloned to the pET11d expression vector (Novagen, Madison, WI, USA).

### 2.2. BceF Protein Expression and Purification for Crystallization

Cultures of transformed *E. coli* BL21(DE3) cells carrying the pET11d vector with the *bceF* gene were grown overnight in Terrific Broth (TB) medium supplemented with ampicillin with shaking at 220 rpm and 37 °C. BceF expression was induced by adding IPTG at 0.1 mM for 20 h at 16 °C. Cells from a four-liter culture were harvested by centrifugation (4500 rpm for 15 min), and resuspended in binding buffer (20 mM imidazole, 0.5 m NaCl, and 20 mM phosphate buffer, pH 7.4). The cells were disrupted by French Press (Cabinet cell disruptor, Constant Systems Ltd., Daventry, UK) at 27 PSI. The cell extract was centrifuged (14,000 rpm for 45 min at 4 °C). The soluble fraction containing the recombinant proteins was purified by Fast Protein Liquid chromatography (FPLC) using the AKTA Avant chromatography system (GE Healthcare, Wauwatosa, WI, USA) equipped with 5 mL HisTrap column (GE Healthcare). The elution buffer contained 0.5 M imidazole, 0.5 M NaCl, and 20 mM phosphate buffer, pH 7.4. The elution was performed by a linear imidazole gradient of 20–500 mM. The fractions containing the protein, as determined with SDS-PAGE (sodium dodecyl sulfate polyacrylamide gel), were pooled together and dialyzed (Spectra/Por^®^, Spectrum Labs, Saint Paul, MN, USA) against Tobacco Etch Virus (TEV) protease activity buffer (50 mM Tris-HCl pH 8, 1 mM DTT, 1 mM EDTA) and TEV protease in order to cleave and remove the 6-His-tag. BceF was then separated from the TEV protease on a 5 mL HisTrap-HP column. The flow through containing the His-tag cleaved BceF was then concentrated using a centrifugal filter device (Amicon Ultra-15, 30 K) and loaded onto a SEC HiLoad 26/600 Superdex 200 column (GE Healthcare). The main protein peak was concentrated to 7.8 mg/mL and stored in 20 mM HEPES, 300 mM NaCl, pH 7.4. Protein concentrations were estimated using a NanoDrop spectrophotometer using extinction coefficients and the molecular weight that were calculated using the ExPASy ProtParam server [15].

### 2.3. BceF Protein Purification for Biochemical Measurements

Bacterial cells from one-liter culture were placed on ice for 15 min, harvested by centrifugation (4500 rpm for 15 min), and resuspended in binding buffer (20 mM imidazole, 0.5 M NaCl, and 100 mM HEPES, 0.05% Tween-20, pH 7.4). The cells were disrupted as described above. The soluble fraction containing the recombinant proteins was purified using 5 mL His-trap as described above. The protein solution was then dialyzed into 0.05 M HEPES, 150 mM NaCl, 4 mM MgCl_2_, 0.02% Tween-20, pH 7.5.

### 2.4. BceF Protein Purification for the Steady-State Kinetics

Bacterial cells from one-liter culture were put on ice for 15 min, harvested by centrifugation (4500 rpm for 15 min), and resuspended in 20 mM imidazole, 0.5 M NaCl, and 100 mM HEPES, pH 7.4. The cells were disrupted as described above. The soluble fraction, containing the recombinant proteins was purified using a 5 mL His-trap column (GE Healthcare) and eluted stepwise.

### 2.5. TEV Protease Expression and Purification

The plasmid was kindly provided by the lab of Prof. David Eisenberg at UCLA, USA. The vector: pDSBA-TEV DsbA mutant/His-tag/TEV protease mutant fusion protein gene (the plasmid description can be found at the following link: http://www.ebi.ac.uk/ena/data/view/EU418501, accessed on 25 June 2016). Cells containing the plasmid were grown overnight on LB plates with ampicillin at 37 °C. A single well-isolated colony was streaked to a new plate and grown for another night. Cells from the plate were used to inoculate TB medium (one-liter shake-flasks) supplemented with ampicillin. The expression of TEV protease was carried out under induction with 1 mM IPTG, which was added when the culture reached OD_600nm_ of ~1.0 at 37 °C, and then the temperature was reduced to 20 °C and expression continued overnight. Cells from one-liter culture were incubated on ice for 15 min, harvested by centrifugation (7000 rpm for 7 min), and resuspended in binding buffer (50 mM Tris pH 8, 0.3 M NaCl). The ice-cooled cells were disrupted twice using a microfluidizer (Tetra Sense, Caesarea, Israel). The cell lysate was centrifuged (32,000 rcf for 15 min at 4 °C), and the soluble fraction containing the recombinant proteins was purified on a 5 mL His-Trap column as described above. The elution buffer contained 0.5 M imidazole, 0.3 M NaCl, and 50 mM Tris buffer, pH 8. The resulting protein fractions were dialyzed into 20 mM Tris pH 8.0, 50 mM sodium chloride, 10% glycerol, 1 mM EDTA, 5 mM β-mercaptoethanol overnight. Further cation purification was performed using a HiTrap S HP column (GE Healthcare) equilibrated with buffer (50 mM sodium chloride, 20 mM Tris pH 8.0). The elution buffer contained 1 M sodium chloride and 20 mM Tris pH 8.0. The protein-containing fractions were pooled and dialyzed against 20 mM Tris pH 8.0, 100 mM sodium chloride, 30% glycerol for several hours, and were then concentrated to 1.5–2 mg/mL. Aliquots were then flash frozen and stored at −80 °C. TEV protease is expressed with a booster protein, which cleaves itself off after expression. The final molecular weight of TEV is estimated to be 28 kDa according to the ExPASy ProtParam web server [15].

### 2.6. BceF Crystallization Experiments

Protein crystallization was carried out using 7.8 mg/mL protein sample by the vapor diffusion method in a 96-well sitting-drop plate. Crystallization drops of 150–300 nL were made by mixing crystallization condition and protein solution at a 1:1 volume ratio, using Mosquito crystallization robot (TTP Labtech, Royston, UK) at the Technion Center for Structural Biology (TCSB). Crystallization conditions were optimized using the Formulator robot (Formulatrix, Bedford, MA, USA). All plates were incubated and regularly imaged in the Rock Imager 1000 robot (Formulatrix) at 293 K. Optimal protein crystals grew in mother liquor containing the reservoir solution of 0.1 M BIS-TRIS, pH 5.5, 25% *w/v* polyethylene glycol 3350 and 3% *v*/*v* 2-Methyl-2,4-pentanediol (MPD). Cryo-protection solutions were made by adding 20% ethylene glycol to the mother liquor solution. Before X-ray data collection, crystals were flash-frozen in liquid nitrogen.

### 2.7. X-ray Data Collection and Structure Determination

We collected X-ray diffraction data at the ID23-EH2 micro-focal beamline of the European Synchrotron Radiation Facility (ESRF). The wavelength of data collection was 0.8729 Å. X-ray data were collected at 100 °K. Reflection indexing, integration, and scaling were performed using XDS/XSCALE [16]. Phases were obtained by molecular replacement using the program Phaser [17] bases on the homologous structure of Etk from *E. coli* (PDB ID 3CIO) [12], which shares 38% sequence identity with the kinase domain of BceF. Crystallographic refinement was performed using Refmac5 [18]. Manual model building was completed in Coot [19] and illustrated with Chimera [20].

### 2.8. Evolutionary Conservation Analysis

Evolutionary conservation scores were calculated using the ConSurf web server [21,22,23] using the BceF kinase domain crystal structure as a query with default parameters.

### 2.9. Crystal Structure Calculations and Visualization

The UCSF Chimera software [20] was used to illustrate the structures in the figures, to construct the BceF octameric model by the alignment of monomeric BceF kinase domain with each of the monomers of Wzc (PDB: 3LA6), for structural comparison between BceF and other BY-kinases dimers and monomers, and to calculate the solved exposed surface areas buried (using the “measure buriedArea” command).

### 2.10. Steady-State Enzymatic Kinetics Analysis of BceF

After purification as mentioned above, the eluted BceF was immediately stabilized by the addition of 1 mg/mL BSA, concentrated to 2.6 mg/mL and flash-frozen for long-term storage in 0.05 M HEPES, 0.5 NaCl, 10% (*v*/*v*) glycerol, pH 7.5. ADP quantification was used to determine BceF phosphorylation activity using two commercial kits employing orthogonal methods for ADP assessment: monoclonal antibody binding (ADP Transcreener, BellBrooks, Madison, WI, USA) and coupled reactions (ADP-Glo, Promega, Madison, WI, USA). For the enzymatic testing, BceF was diluted in kinase activity buffer (50 mM HEPES pH 7.5, 150 mM NaCl, 4 mM MgCl_2_, 0.01% (*v*/*v*) Triton X-100, 1% (*v*/*v*) dimethyl sulfoxide (DMSO)). Kinetic parameters for BceF were measured by both Transcreener fluorescence intensity and ADP-GloTM luminescence-coupled assay at ambient temperature at pH 7.5. The reaction was initiated by the addition of BceF enzyme to a final concentration of 100 µg/mL in triplicate. The plates were shaken in the ClarioStar BioTech plate reader for up to 75 min. At the designated time points, the reaction was quenched by the addition of ethylenediaminetetraacetic acid (EDTA) (Transcreener) or ADP-Glo buffer (ADP-Glo). Downstream steps were performed according to the manufacturer’s instructions. The ADP content was determined by running the respective ATP-to-ADP conversion plots. The ADP content was then plotted versus the experiment time frame to calculate the slope of the linear portion representing the initial velocity. The velocity was then plotted as a function of ATP concentration in GraphPad Prism6 and the data were subjected to non-linear regression analysis (Enzyme kinetics, Michaelis-Menten analysis in the software).

### 2.11. MST Measurement of the Affinity of BceF for the ATP Analog

Adenylyl-imidodiphosphate (AMP-PNP) (Roche Diagnostics GmbH, Basel, Switzerland10102547001) stock solution was prepared in ultra-Pure Water (Biological Industries, Beit HaEmek, Israel) at a concentration of 50 mg/mL. Tween-20 was added to obtain a stock of 98.77 mM AMP-PNP and 0.05% Tween-20. This solution was serially diluted for the MST testing in double-distilled water (DDW) with 0.05% Tween-20 and then mixed with the labeled protein at a 1:1 ratio. BceF was labeled using Monolith NT™ Protein Labeling Kit RED-NHS using NT-647 (RED) fluorescent dye (Nano Temper Technologies, Munich, Germany). The labeled BceF was stored in 0.05 M HEPES, 150 mM NaCl, 4 mM MgCl_2_, 0.02% Tween-20, pH 7.5 at −80 °C. Labeled BceF concentration was determined to be 13.08 µm using Equation (1) and the NT-647 (RED) florescent dye characteristics and BceF characteristics. For the MST measurements, BceF was diluted to 1:25 in the activity buffer and distributed at a 1:1 ratio with the serial dilution of the tested ligand AMP-PNP. The labeled protein concentration after 1:50 dilution was 0.26 µm. The labeled BceF was mixed with AMP-PNP serial dilutions and incubated in the dark at room temperature for 1 h before being measured in the Monolith™ NT.115 MST device (Nano Temper Technologies) at 23.11 °C using 20% excitation at 20% power using Monolith™ NT.115 MST premium coated capillaries. Three replicates of each concentration were analyzed using the MO. Affinity Analysis software.

Equation (1): the equation provided by NanoTemper to calculate the concentration of labeled protein.

(1) $$C_{Prot}=\frac{A_{Prot}}{{Ɛ}_{Prot}d}=\frac{A_{280}-A_{max}CF}{{Ɛ}_{Prot}d}$$

NT-647 (RED) characteristics: λmax = 650 (nm), εmax = 250,000 (M^−1^ cm^−1^), CF = 0.027, BceF extinction coefficient: ε = 10,430 (M^−1^ cm^−1^).

### 2.12. Size Exclusion Chromatography-Multi-Angle Light Scattering (SEC-MALS)

The size exclusion chromatography (SEC) was performed using a Superdex 75 10/300 SEC column operated by AKTA Avant (GE Healthcare). The multi-angle light scattering (MALS) was performed with miniDAWN TREOS (WYATT Technology) and its companion Optilab T-rEX (WYATT Technology, Santa Barbara, CA, USA) dRI detector. The analysis of the SEC-MALS data was performed using the ASTRA software (WYATT Technology).

### 2.13. Tandem Mass Spectrometry (MS/MS) Analysis

The purified BceF was resolved by SDS-PAGE, excised from the gel, subjected to the digest with trypsin and endoproteinase Lys-C, and analyzed by liquid chromatography with tandem mass spectrometry (LC–MS/MS) at the Smoller proteomics center at the Technion-Israel institute of technology.

## 3. Results and Discussion

### 3.1. The Crystal Structure of the BceF Kinase Domain

**Figure 1:**
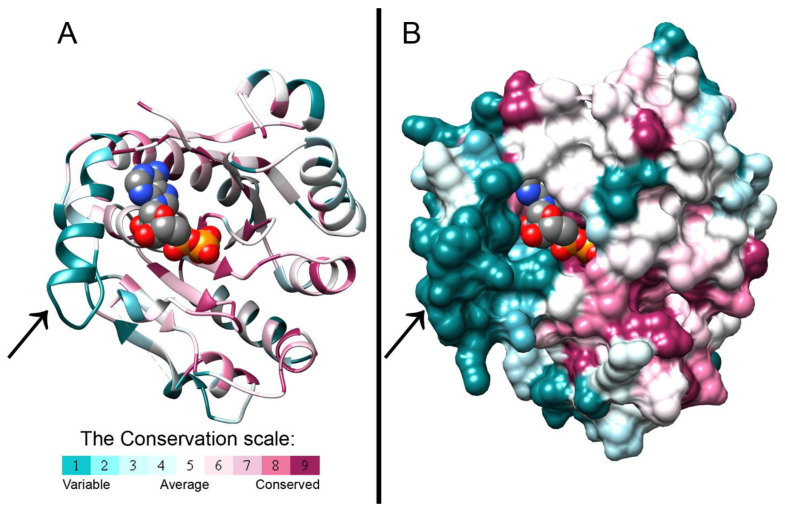
Evolutionary conservation scores projected on the crystal structure of the BceF kinase domain. The crystal structure of the BceF kinase domain (showing one monomer) is colored by evolutionary conservation scores calculated using the ConSurf webserver [21,22,23]; a variable-to-conserved colored scale is indicated. ADP bound at the active site is represented as space-filled atoms and colored by atom-type with carbons in grey, oxygen in red, nitrogen in blue, and sulfur in yellow. BceF is shown either as ribbons to indicate secondary structures (**A**) or in a solvent-accessible surface representation (**B**). An evolutionary variable helix (residues 500–511, with the exception of the conserved Gln502) and the following evolutionary variable loop (residues 512–518) close to the active site are indicated with an arrow.

**Figure 2:**
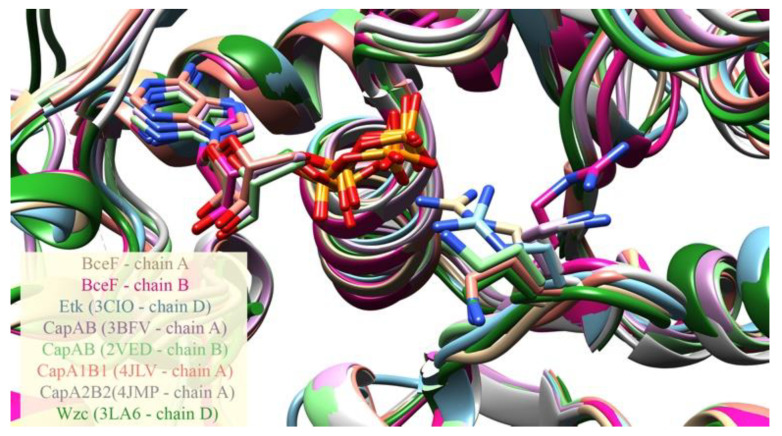
Conformational variability of the ADP-proximal residue Arg590 in BceF and its equivalent positions in other BY-kinases. ADP is present in both BceF chains, and CapAB (PDB Ids 3BFV, 2VED and 4JLV). ADP carbons are colored the same as the backbone of the protein from the same determined structure. The figure depicts a structural superimposition of the kinase domain of several BY-kinases, and of the two chains in the asymmetric unit in the crystal structure of BceF, with zoom-in into the ADP ligand and into the Arg590 residue in BceF and the equivalent positions in the other BY-kinases, shown as sticks. Side-chain atoms are colored by atom types (nitrogen in blue, oxygen in red and sulfur in yellow). The backbone and carbon atoms in each BY-kinase and BceF chain are colored differently: BceF chain A in beige, BceF chain B in magenta, Etk (PDB code 3CIO) in cyan (featuring the equivalent Arg572), CapAB (PDB code 3BFV in purple, PDB code 2VED in light green, PDB code 4JLV in orange, PDB code 4JMP in grey), featuring the equivalent Lys1082, and Wzc (PDB code 3LA6 in dark green), featuring the equivalent Lys567.

The crystal structure of *B. cepacia* BceF kinase domain, overexpressed and purified from *E. coli*, was determined at 1.85Å resolution (Figure 1 and Table 1). The plasmid construct used encompassed the BceF kinase domain, i.e., residues 471–741. All residues were resolved in the electron density map except for residues 471–476, 598–602, and 723–741. These regions are not evolutionary conserved and likely correspond to unstructured regions. The structure features a fold similar to other BY-kinases of known structures, showing a central, eight-stranded curved β-sheet, flanked by 12 helices of varying lengths (Figure 1). In the crystallographic asymmetric unit, there are two virtually identical kinase domains of BceF, with a root-mean-square deviation (RMSD) of 0.36 Å across 241 paired residues (Figure S2). The structure revealed an adenosine diphosphate (ADP) molecule bound at the active site, probably co-purified from the bacteria, since it was not added during the purification or crystallization of BceF (Figure 1, Figure S2). The catalytic pocket exhibits the ATP-binding motifs common to BY-kinases and other nucleotide-binding proteins, including Walker A (residues _556_GPTPGIGKS_564_), Walker A’ (residues _585_DAD_587_), and Walker B (residues _661_VVLID_665_). Evolutionary conservation analysis showed that the nucleotide-binding pocket is generally conserved in BY-kinases, with yet few exceptions of moderately conserved and evolutionary variable regions (Figure 1). The most evolutionary variable region around the active site comprises a helical region in BceF (residues 500–511, with the exception of the conserved Gln502) and the following evolutionary variable loop (residues 512–518) (marked with an arrow in Figure 1). The active site pocket in BceF, with side chains closer than 4 Å to the ADP molecule, includes the highly conserved Pro497, Ser499, Gln502, Ile561, Lys563, Ser564, Phe565, Arg590, and Asn719 residues, along with the moderate evolutionary conserved Gln498, Pro559, Arg690, and the evolutionary variable Asp506 and Arg517 residues. Of note, Arg590 is facing the active site only in one of the BceF molecules in the asymmetric unit (chain A), but not in the other. The equivalent residue in other tyrosine kinases indeed shows relative flexibility (Figure 2). This observation is surprising given that the putative role of this positively charged position is to stabilize the adenosine phosphate group at the active site [3]. It is not clear if this variability represents an artifact of crystallization or constitutes a regulatory phenomenon.

### 3.2. Evolutionary Conservation Analysis and Structural Comparison of BY-Kinases

**Figure 3:**
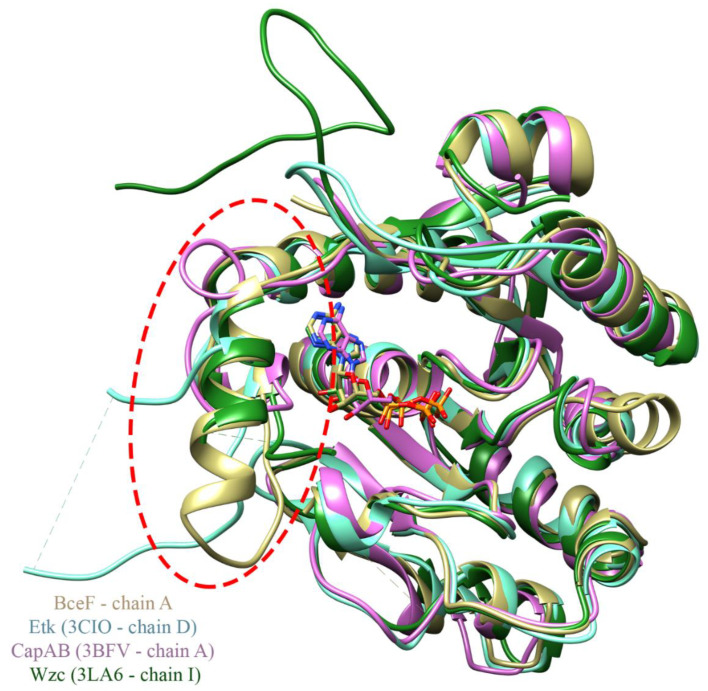
Structural superimposition of the BceF, Etk, CapA/B, and Wzc kinase domains. Ribbon diagrams of the structure of BceF from *B. cepacia* colored in beige; Etk from *E. coli* in cyan (PDB: 3CIO [12]); CapA/B from *S. aureus* is purple (PDB: 3BFV [14]); Wzc from *E. coli* in dark green (PDB: 3LA6 [11]). Dashed lines represent regions that could not be resolved in the respective crystal structures. ADP, present in the crystal structure of BceF, CapA/B, and Wzc, is shown in balls and sticks and is colored by atom-type (beige, purple, and dark green carbons for the ADP from BceF, CapA/B, and Wzc, respectively). An evolutionary variable region (Figure 1) that is also structurally different between the kinases is evident on the utmost left side of the structures in this view, marked by a dashed red circle. The RMSD between BceF and Etk is 0.881 Å for 196 successfully aligned atom pairs, or “pruned” by the default criteria in Chimera [20], and 3.305 Å for all 238 pairs. The RMSD between BceF and CapA/B is 1.100 Å for 186 pruned atom pairs and 3.867 Å for all 232 pairs, and between BceF and Wzc is 0.885 Å for 208 pruned atom pairs and 1.930 Å for all 235 pairs.

Structural comparison between the kinase domain of BceF and that of Etk from *E. coli* [12], CapA/B from *S. aureus* [14], and Wzc *E. coli* [11], shows a conserved overall fold of the central curved β-sheet flanked by helices, with some variation in the length of the secondary structure elements. Intriguingly, the most significant differences between the three BY-kinases lie in the region near the active site (Figure 3). This corresponds to the evolutionary non-conserved helix-loop region in BceF (residues 500–519). The equivalent region in Etk displays a much shorter helix (residues 474–477) followed by a longer loop (residues 478–501), and part of it was not resolved in the EtK crystal structure (residues 483–491). The corresponding region in the CapA/B chimera is occupied by a short loop located at the chimeric constructed linker between the fragment from the C-terminus of the associated transmembrane adaptor and the N-terminus of the cytoplasmic BY-kinase (residues 222, 1001–1012) (Figure 3). The corresponding region in Wzc is composed of a helix (residues 473–481) and a loop (residues 482–496) that is partly unresolved (residues 483–491). This 24-residue region in Wzc is called the RK-cluster due to its positively charged arginine- and lysine-rich region (two arginine and four lysine residues), and it was suggested that the helix in the RK-cluster is important for the kinase activity, while the flexible region may be a regulatory domain with a role in capsular polysaccharide synthesis [11]. The equivalent 20-resdiue region in BceF (residues 500–519) contains two arginine and two lysine residues. Overall, this region is distinctly different in BY-kinases with known structures both at the primary and secondary structures (Figure 1) and may fulfill a regulatory role. The proximity of this helical/loop region to the active site of the kinase suggests that it confers substrate specificity varying across bacterial strains. It is therefore likely that molecules designed to bind within this region and to block the active site may offer a strain-specific antivirulence therapeutic approach.

### 3.3. Steady-State Kinetics Reveals Low Enzymatic Efficiency of the BceF Kinase Domain

BY-kinases are known to undergo autophosphorylation on the tyrosine-rich C-terminal region [12]. To characterize the natural phosphorylation pattern of BceF, we used tandem mass spectrometry (MS/MS) to determine the prevalence of the PO_4_^−3^ modification at each of the three tyrosines in a peptide with the sequence _725_ARG**Y**GRG**Y**AAVHE**Y**LSA_741_ corresponding to the BceF C-terminal region. Three main populations are presented in (Figure S3). The non-phosphorylated population comprised 28% of the peptides. The second population with monophosphorylation, mostly of the third tyrosine at position 14, comprised 68% of the peptides, while only minor phosphorylation was observed at positions 4 and 8 (1.6% and 0.4% of the peptide, respectively). The remaining two percent were peptides di-phosphorylated at positions 8 and 14. Thus, the phosphorylation of the C-terminal tail occurs predominantly at the distal tyrosine residue of the C-terminal region. We, therefore, chose to conduct enzymatic characterization using a shorter peptide from the tyrosine-rich region containing the third distal tyrosine only (_733_AAVHE**Y**LSA_741_).

**Figure 4:**
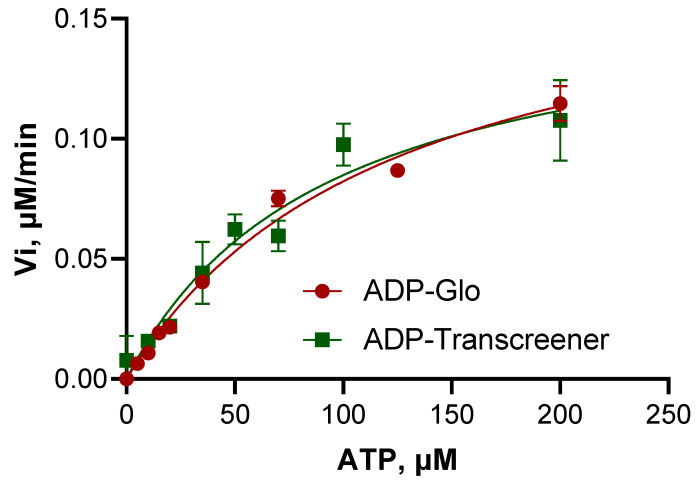
Michaelis–Menten plots of BceF kinase domain.

BceF kinase activity was assessed using steady-state kinetics data fitted to the Michaelis–Menten model, carried out in parallel using two different assays: (1) monoclonal antibody binding method using Transcreener^®^ ADP FI (BellBrooks Labs) and (2) coupled reactions method using ADP-Glo^TM^ kinase assay (Promega). The steady-state kinetics parameters determined from both assays were rather similar, with V_max_ values of 0.1634 and 0.1835 µM min^−1^, and K_m_ values (for ATP) of 92 and 123 µM, for the Transcreener assay and for the ADP-Glo^TM^ kinase assay, respectively (Figure 4, Table 2). The K_cat_ turnover rate constant was 0.0481 min^−1^ for the Transcreener assay and 0.0541 min^−1^ for the ADP-Glo^TM^ kinase assay (Table 2), indicating that BceF phosphorylation catalysis is rather slow at least for the tested substrates. The corresponding K_cat_/K_m_ values for the respective assays were 5.2 × 10^−4^ and 4.4 × 10^−4^ min^−1^ µM^−1^, indicating relatively low enzymatic efficiency for the substrate tested. As a reference, the protein tyrosine kinase P72^syk^ purified from rat spleen exhibited K_cat_/K_m_ values ranging from 0.01 to 18.5 min^−1^ µM^−1^ for a variety of synthetic peptides representing naturally occurring phospho-acceptor sites [27].

Michaelis–Menten plots of the isolated BceF kinase domain measured by Transcreener^®^ and ADP-Glo^TM^ assays for the phosphorylation of the _733_AAVHE**Y**LSA_741_ peptide.

### 3.4. BceF Kinase Domain Displays a Low Binding Affinity for the Non-Hydrolyzable ATP Analog AMP-PNP

**Figure 5:**
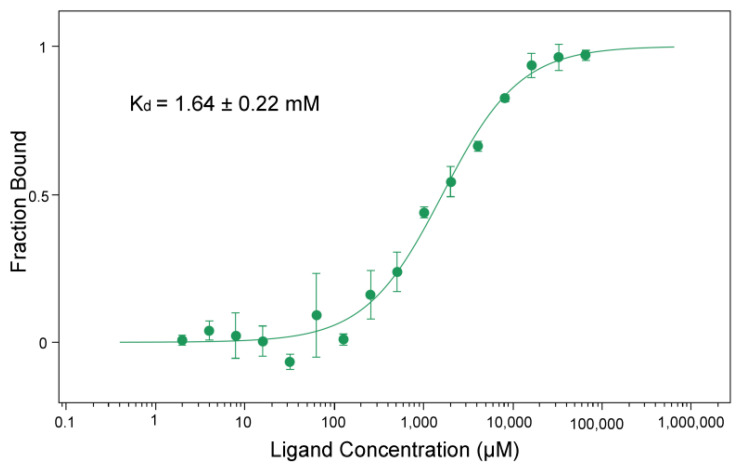
Binding affinity of AMP-PNP for BceF kinase domain.

MicroScale Thermophoresis (MST) was used to evaluate the binding affinity of BceF for Adenylyl-imidodiphosphate (AMP-PNP), a non-hydrolyzable analog of ATP. The measured dissociation constant K_d_ was 1.64 ± 0.22 mM (Figure 5). The affinity of tyrosine kinase Wzc from *E. coli* for MANT-ADP, an analog of ADP, was Kd = 0.59 ± 0.12 µM [11]; however, the two compounds are quite different and cannot be directly compared.

The binding of AMP-PNP to BceF kinase domain was quantified using microscale thermophoresis. The protein-bound fraction is plotted against AMP-PNP concentration. Error bars correspond to the standard deviation for three replicates. The standard error of the regression was 0.93.

### 3.5. BceF Kinase Domain Exists as a Dimer in Solution and in the Crystals

**Figure 6:**
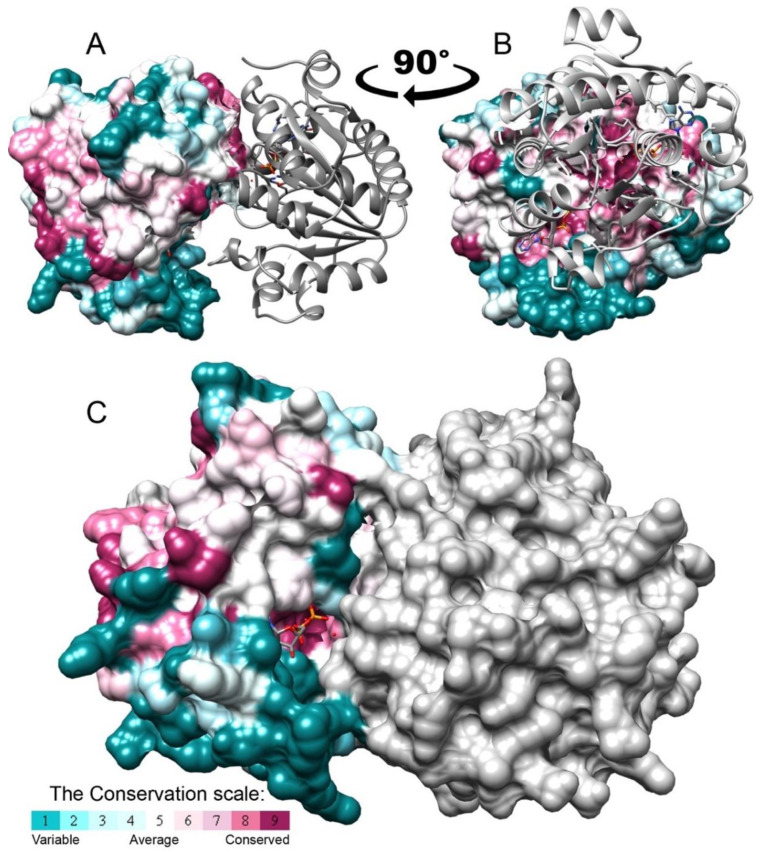
The isolated BceF kinase domain forms a dimer in the crystal structure. A dimer of the BceF kinase domain was observed in the crystal structure, demonstrating a partial obstruction of the active site. One monomer is colored grey and the other by evolutionary conservation scores calculated using the ConSurf web server [21,22,23]; a variable-to-conserved colored scale is indicated. ADP, present in the crystal structure, is shown in format of sticks and colored by atom type. (**A**,**B**) Two views (representing −90° rotation along the vertical direction) of the BceF dimer with one monomer displayed as ribbons and the other as solvent-accessible surface representation. (**C**) The two monomers are showed using a solvent-accessible surface representation.

We examined the oligomerization state of BceF kinase domain in solution using size exclusion chromatography with multi-angle light scattering (SEC-MALS) [28,29]. The analysis revealed a single oligomeric species with a molecular mass of 59.1 kDa (±5.45%) (Figure S5). Since the monomeric mass of the BceF kinase domain calculated based on the sequence is 29.4 kDa, the SEC-MALS analysis supports the predominant dimeric state of the BceF kinase in solution. Consistently, the BceF kinase domain crystallized as a dimer in the asymmetric unit (Figure 6). Similarly, previously determined structures of BY-kinases also showed a dimeric arrangement, including Etk from *E. coli* ([12] PDB code 3CIO) and CapA/B from *S. aureus* ([14], PDB code 3BFV and 2VED). An octameric arrangement was obtained using active-site mutations that putatively prevented autophosphorylation and disassembly of the octamer of Wzc from *E. coli* ([11] PDB code 3LA6), and of CapA/B from *S. aureus* ([14] PDB code 2VED). It was thus suggested that BY-kinases function as octamers [14].

We accordingly generated an in silico model of an octameric form of BceF based on the structures of the tyrosine kinase Wzc K540M mutant from *E. coli* ([11] PDB code 3LA6) by a structural alignment of BceF monomers with each of the monomers of Wzc. The interface between monomers in the modeled octameric structure is indeed evolutionarily conserved within BY-kinases (Figure S4), supporting the biological relevance of this octameric configuration. In contrast, the interface of the dimer observed in the crystal packing of the BceF kinase domain was not evolutionary conserved (Figure 6A,B). Moreover, in this dimeric arrangement, access to the active site is partially blocked (Figure 6C). Figure S6 shows the different interfaces between the subunits of the dimer determined in the crystal structure and the modeled octamer. This face-to-face configuration may suggest a non-physiological dimeric arrangement as a result of a crystalization artifact, or possibly due to the absence of other cytoplasmic or membranal BceF domains in the crystallized construct. This is consistent with the relatively small buried solvent surface area of 789 Å^2^ at the dimeric interface. The crystallographic dimers of active Etk *E. coli* ([12] PDB code 3CIO) and CapA/B ([14], PDB code 3BFV) displayed a solvent surface area buried of 958 Å^2^ and 603 Å^2^ at the dimeric interface, respectively. For comparision, in the putatively active octamer of CapA/B ([14], PDB code 2VED), the solvent surface area buried is 1027 Å^2^ and 1141 Å^2^ for the interfaces in each side of the monomer. A structural alignment shows that each kinase assumes a different dimeric configuration (Figure S7). Furthermore, the dimeric interface of both CapAB and Etk is not conserved (Figures S8 and S9). This might be the result of the self-assembly tendency of the kinase domains lacking constraints to the transmembrane portions of the native kinase. Alternatively, this dimer may represent a latent oligomeric arrangement that requires conformational changes and rearrangement for maximal enzymatic activity. If the BceF kinase domain dimer observed in solution has the same arrangement observed in the crystal form, the partial obstruction of the active sites by the interface might explain the low enzymatic efficiency observed. The latter postulation should be taken with some caution since the crystallization and the SEC analysis were carried out at different pHs (5.5 vs. 7.5, respectively), which could potentially give rise to structurally different dimers.

## 4. Conclusions

The rapid increase in the occurrence of antibiotic resistance by many common bacterial pathogens [30] calls for urgent development of new classes of antibiotics [4]. BY-kinases are promising targets for novel antibacterial agents, primarily due to their importance in extracellular polysaccharide synthesis and virulence [3,14]. BceF from the pathogenic *B. cepacia* [2] is involved in the production of exopolysaccharides of the biofilm matrix, which acts as a barrier for drug delivery and thus increases bacterial resilience and resistance to modern antibiotics [31]. We have determined the crystal structure of the kinase domain of BceF and studied its enzymatic activity. We found that the BceF kinase domain forms dimers in solutions and in crystals, which may not represent the physiological quaternary structure of the entire protein. The partially obstructed active sites of this dimer may explain the relatively low binding affinity of the isolated kinase domain of BceF for an ATP analog and the low catalytic efficiency measured in solution. The reported structure of the BceF kinase domain provides an experimental structural framework for structure-based drug design of compounds with the potential to specifically inhibit BceF and biofilm formation by *Burkholderia cepacia*. For instance, the structure can be used for the designing of compounds to bind and block the active site (around the bound ADP). Compounds with the capacity to also interact with the non-conserved helix-loop region adjacent to the active site may provide increased specificity for the inhibition of BceF over other BY-kinases.

## Figures and Tables

**Table 1:** Data collection and refinement statistics (molecular replacement) of BceF kinase domain.

	BceF
PDB accession code	6Z0P
Beamline	ESRF ID23-2
Date	25 June 2016
Data collection	
Space group	P 1 21 1
Cell dimensions	
*a*, *b*, *c* (Å)	44.27 90.48 61.34
α, β, γ (°)	90.0 111.1 90.0
Wavelength (Å)	0.8729
Resolution (Å)	90.5-1.85 (2.2-1.85)
R-factor observed (%)	9.5 (47.3)
^a^*R*_meas_ (%)	11.2 (55.1)
*I* / sigma	9.6 (2.7)
Total reflections	142,307 (58,228)
Unique reflections	38,309 (15,478)
Completeness (%)	99.4 (99.5)
Multiplicity	3.7 (3.8)
^b^ CC_1/2_ (%)	99.7 (80.6)
Refinement	
Resolution (Å)	48.4-1.85 (1.90-1.85)
Completeness (%)	99.3 (96.6)
^c^ No. reflections	36413
^d^*R*_work_ (%)	18.3 (25.6)
*R*_free_ (%)	20.2 (23.5)
*R*_free_ value test set size (%)	5
No. atoms	3780
Protein	1836 (Chain A; 271 residues) 1839 (Chain B; 271 residues)
Ligand/ion	54 (ADP)
Water	51
*B*-factors	
Protein	26.3 (Chain A) 24.8 (Chain B)
Ligand/ion	20.6 (ADP)
Water	24.8
R.m.s. deviations	
Bond lengths (Å)	0.005
Bond angles (°)	1.272
Clash score *	4.6 (97th percentile)
Molprobity score *	1.24 (99th percentile)
Number of xtals used for scaling	1

Values in parentheses are for highest-resolution shell. (^a^) R-meas is a redundancy-independent R-factor defined in Reference [24]. (^b^) CC_1/2_ is the percentage of correlation between intensities from random half-datasets [25]. (^c^) Number of reflections corresponds to the working set. (^d^) Rwork corresponds to working set. * Calculated using Molprobity [26].

**Table 2:** Calculated kinetic parameters for BceF kinase domain.

	ADP-Glo	Transcreener
V_max_, μM min^−1^	0.1835 ± 0.0107	0.1634 ± 0.0198
K_m_, μM	123.06 ± 13.78	92.51 ± 22.58
K_cat_, min^−1^	0.0541 ± 0.0031	0.0481 ± 0.0058
K_cat/_K_m_, μM^−1^ min^−1^	0.0004	0.0005

Calculated Michaelis–Menten parameters for the phosphorylation of the _733_AAVHE**Y**LSA_741_ peptide by the isolated BceF kinase domain using the Transcreener^®^ and ADP-Glo^TM^ assays. V_max_ and K_m_ are represented as means ± standard errors of three replicates.

## Data Availability

The coordinates of the octameric model are available upon request.

## Supplementary Materials

The following are available online at https://www.mdpi.com/article/10.3390/biom11081196/s1, Figure S1: Schematic organization of bacterial tyrosine kinases, Figure S2: Electron density map around the active site of the BceF kinase domain, Figure S3: Phosphorylation states of the tyrosine-rich tail of BceF, Figure S4: A constructed structural model of BceF kinase domain octamer, Figure S5: SEC-MALS chromatogram of purified BceF, Figure S6: The interface of the BceF octamer model versus the BceF dimer, Figure S7: Structural alignment of crystallographic dimers of BY-kinases, Figure S8: The isolated CapAB kinase domain forms a dimer in the crystal structure, Figure S9: The isolated Etk kinase domain forms a dimer in the crystal structure.
