# Supplementary material for: Structural and Functional Insights into the Biofilm-Associated BceF Tyrosine Kinase Domain from Burkholderia cepacia
Source: Biomolecules. 2021 Aug 12;11(8):1196. doi: 10.3390/biom11081196 (PMC8394532; doi:10.3390/biom11081196)
Supplement: Supplementary file 1 [file biomolecules-11-01196-s001.zip › biomolecules-1315734-supplementary.pdf]

Supplementary Figures

Structural and Functional Insights into the Biofilm-associated BceF Tyrosine Kinase Domain from *Burkholderia cepacia*

Michal Mayer, Yulia Matiuhin, Mickal Nawatha, Orly Tabachnikov, Inbar Fish, Nili Schutz, Hay Dvir*, and Meytal Landau*

Figure S1. Schematic organization of bacterial tyrosine kinases

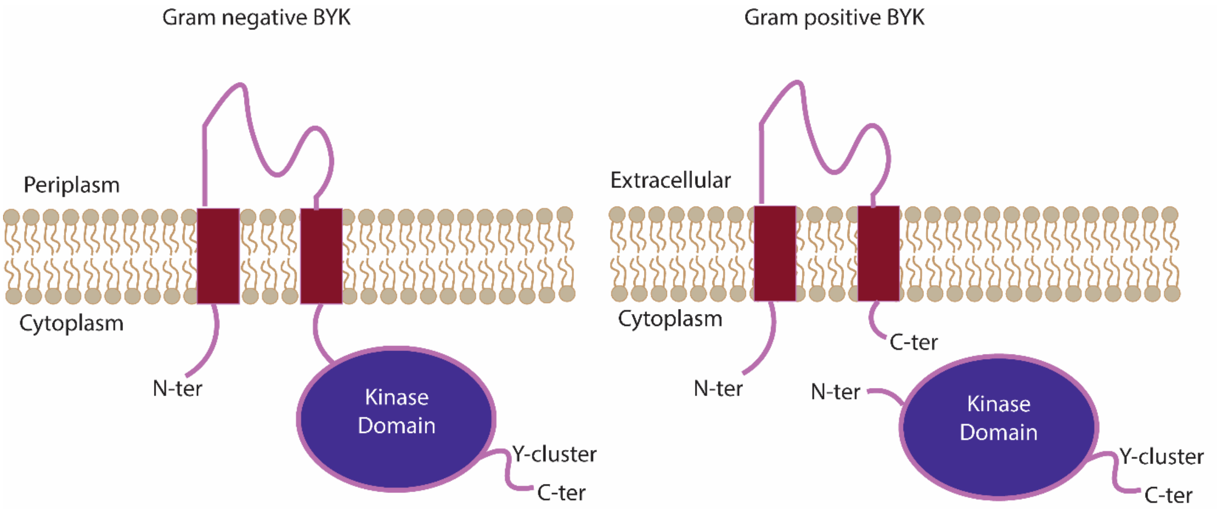

Schematic organization of bacterial tyrosine kinases in Gram negative (left) and Gram positive (right) bacteria. BY kinases share a catalytic, intracellular kinase domain. At the C-terminal end of the BY kinases is a Tyrosine rich area named the Y-cluster.

Figure S2. Electron density map around the active site of the BceF kinase domain

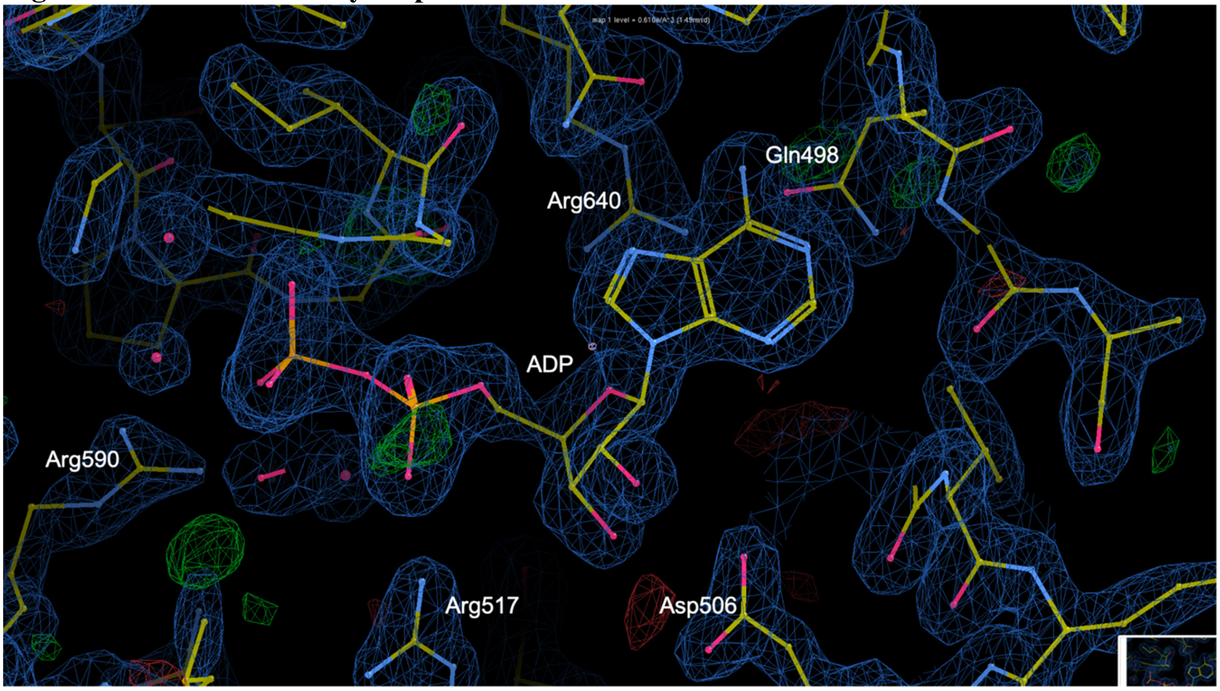

Difference electron density maps (2Fo – Fc; blue at 1.5 sigma and Fo – Fc, green positive at 3.0 sigma) around the active site of chain A in the structure of the BceF kinase domain. Residues surrounding the ADP are indicated in sticks model. The image was generated using Coot [29]

Figure S3. Phosphorylation states of the tyrosine-rich tail of BceF

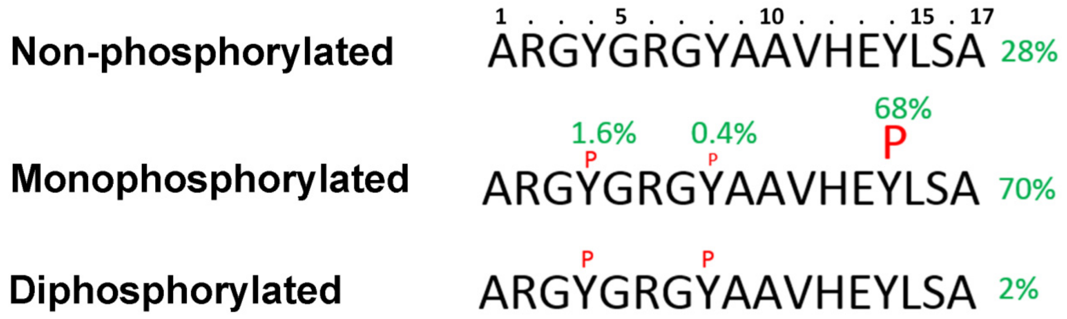

The prevalence of each potential phosphorylation along a tyrosine-rich peptide corresponding to the C-terminal region of BceF kinase domain (residues 725-741) was analyzed using liquid chromatography with tandem mass spectrometry. The red "P" designates the phosphorylation positions, whereas its size is proportional to the occupancy of the specific phosphorylation site. The green numbers indicate the percentage of the indicated phosphorylation state among all possible states analyzed.

Figure S4. A constructed structural model of BceF kinase domain octamer

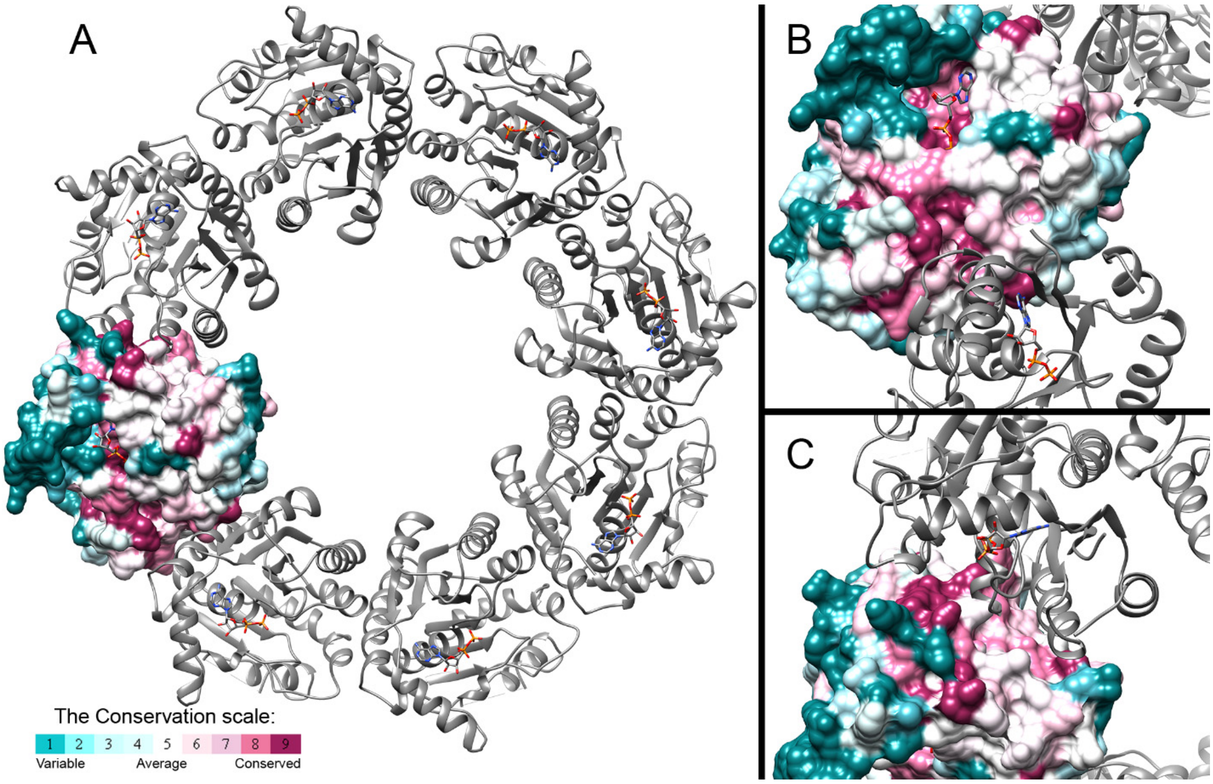

The model of the octameric state of BceF kinase domain was obtained by superimposition of BceF monomers into the octameric structure of the Wzc K540M mutant from *E. coli* ([10] PDB code 3LA6). BceF is presented as grey ribbons except for one of the monomers for which the solvent accessible surface is colored by evolutionary conservation scores calculated using the ConSurf webserver [14–16]; a variable-to-conserved colored scale is indicated. ADP, present in the crystal structure, is shown in sticks format colored by atom-type. (A) The octameric assembly. (B-C) A zoom-in view into the two interfaces between monomers in the octameric model, indicating high evolutionary conservation of interface residues.

Figure S5. SEC-MALS chromatogram of purified BceF

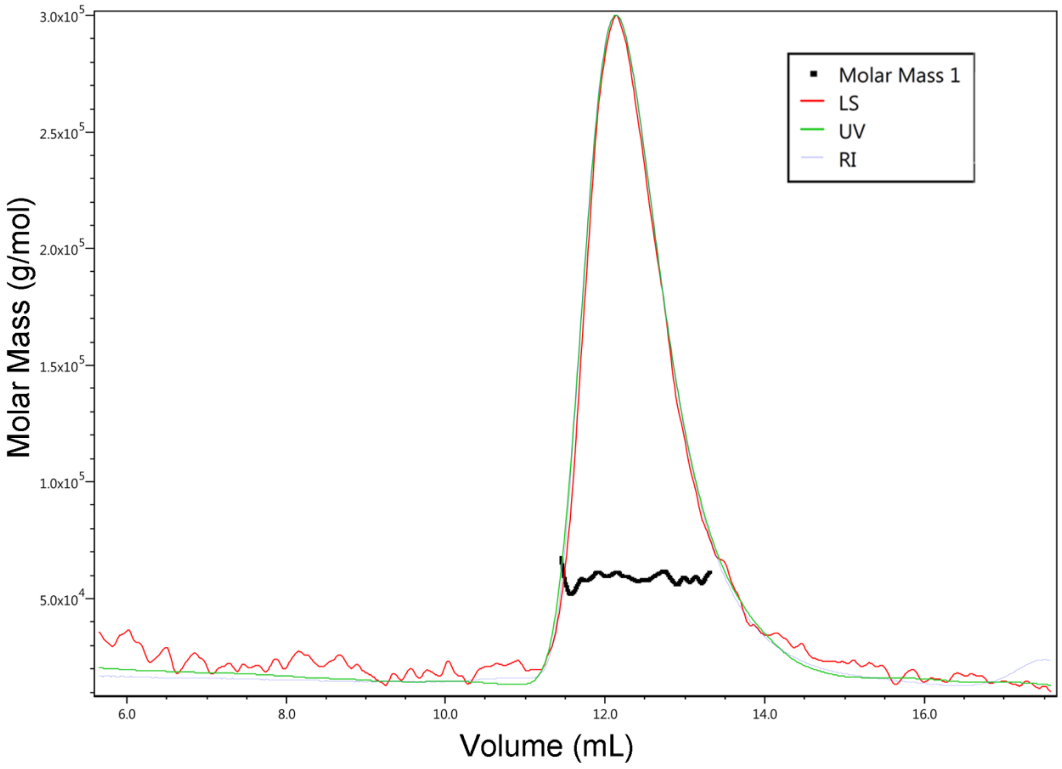

SEC-MALS chromatogram of the elution of BceF of the Superdex™ 75 10/300 GL analytical column showing one main peak. The molecular mass of BceF, as determined by SEC-MALS, was 59130 Da (±5.45%) and corresponds to the size of a dimer (the theoretical monomeric mass is 29428.9 Da.) The graph was extracted from ASTRA software.

Figure S6. The interface of the BceF octamer model versus the BceF dimer

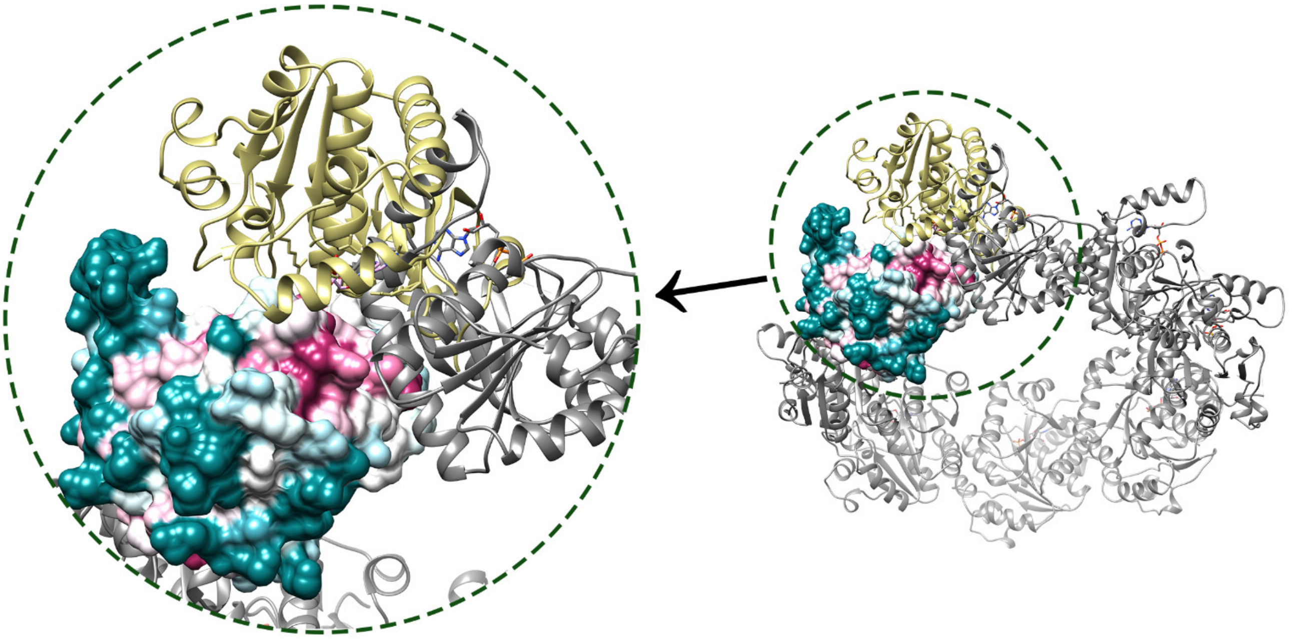

One monomer of the BceF kinase domain dimer observed in the crystal structure (beige) is structurally aligned to one monomer of the constructed model of the octameric state of BceF (grey). The interface region of the dimeric and octameric subunits is enlarged (circle). The aligned chain shared by the dimer and the octamer is shown with a solvent accessible surface colored by evolutionary conservation scores calculated using the ConSurf webserver [14–16]. ADP, present in the crystal structure, is shown in sticks format colored by atom-type.

Figure S7. Structural alignment of crystallographic dimers of BY-kinases

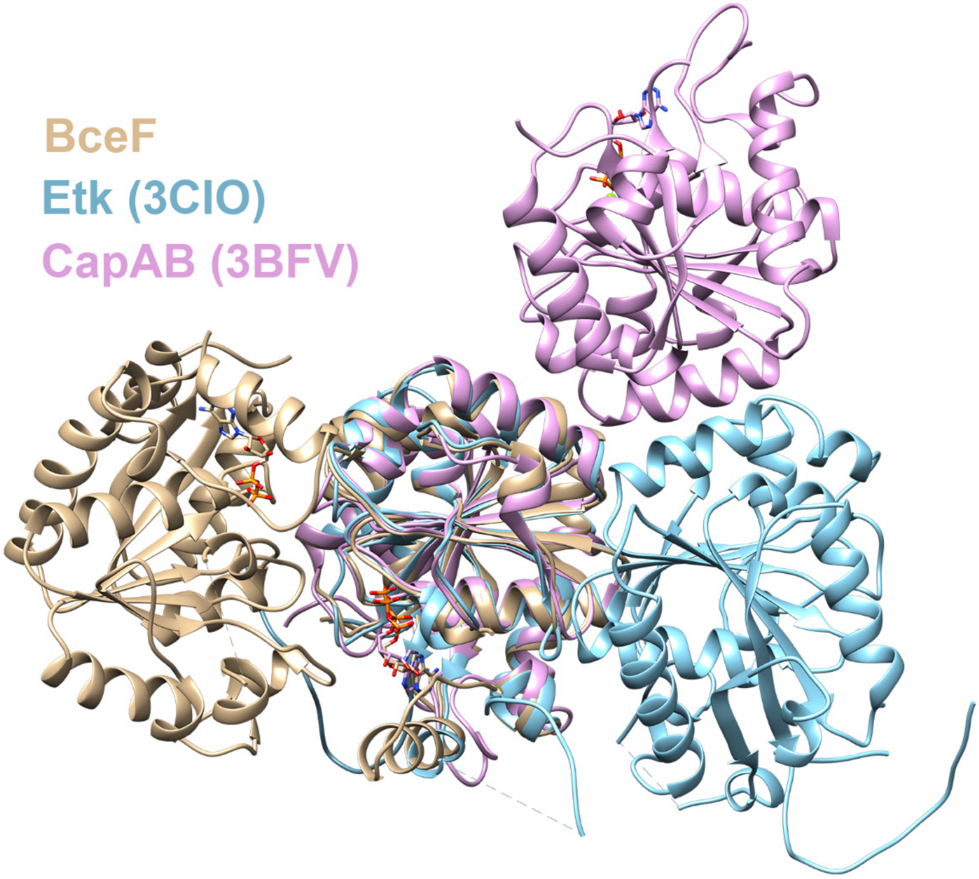

The figure depicts structural superimposition of crystallographic dimers of three BY-kinases. BceF from *B. cepacia* is presented as beige ribbons, Etk from *E. coli* is presented as blue ribbons ([11] PDB code 3CIO), CapAB from *S. aureus* is presented as purple ribbons ([13] PDB code 3BFV). Dashed lines represent protein segments that could not be resolved in the crystal structures. The bound ADP molecules in the BceF and CapAB structures, are shown in sticks format colored by atom-type.

Figure S8. The isolated CapAB kinase domain forms a dimer in the crystal structure

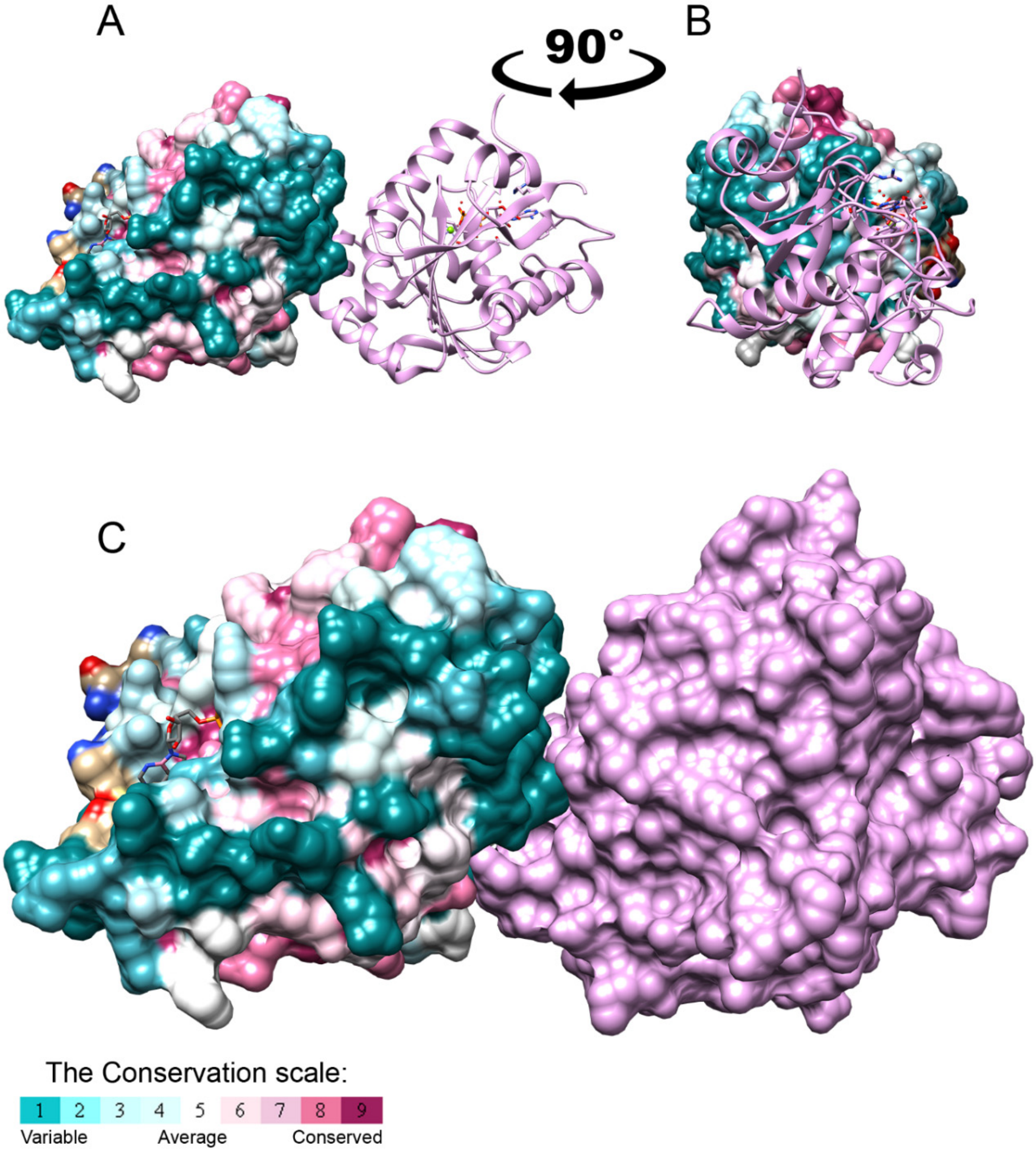

The CapAB kinase domain forms a dimer in the crystal structure (PDB id 3BFV). One monomer is colored purple and the other is colored by evolutionary conservation scores calculated using the ConSurf webserver [14–16]; a variable-to-conserved colored scale is indicated. A region of the CapAB chimera, which is not part of the multiple sequence alignment is in brown carbons. ADP, present in the crystal structure, is shown in sticks format and colored by atom-type. (A-B) Two views (representing ~90° rotation along the vertical direction) of the CapAB dimer with one monomer displayed as ribbons and the other as solvent-accessible surface representation. (C) The two monomers are showed using a solvent-accessible surface representation.

Figure S9. The isolated Etk kinase domain forms a dimer in the crystal structure

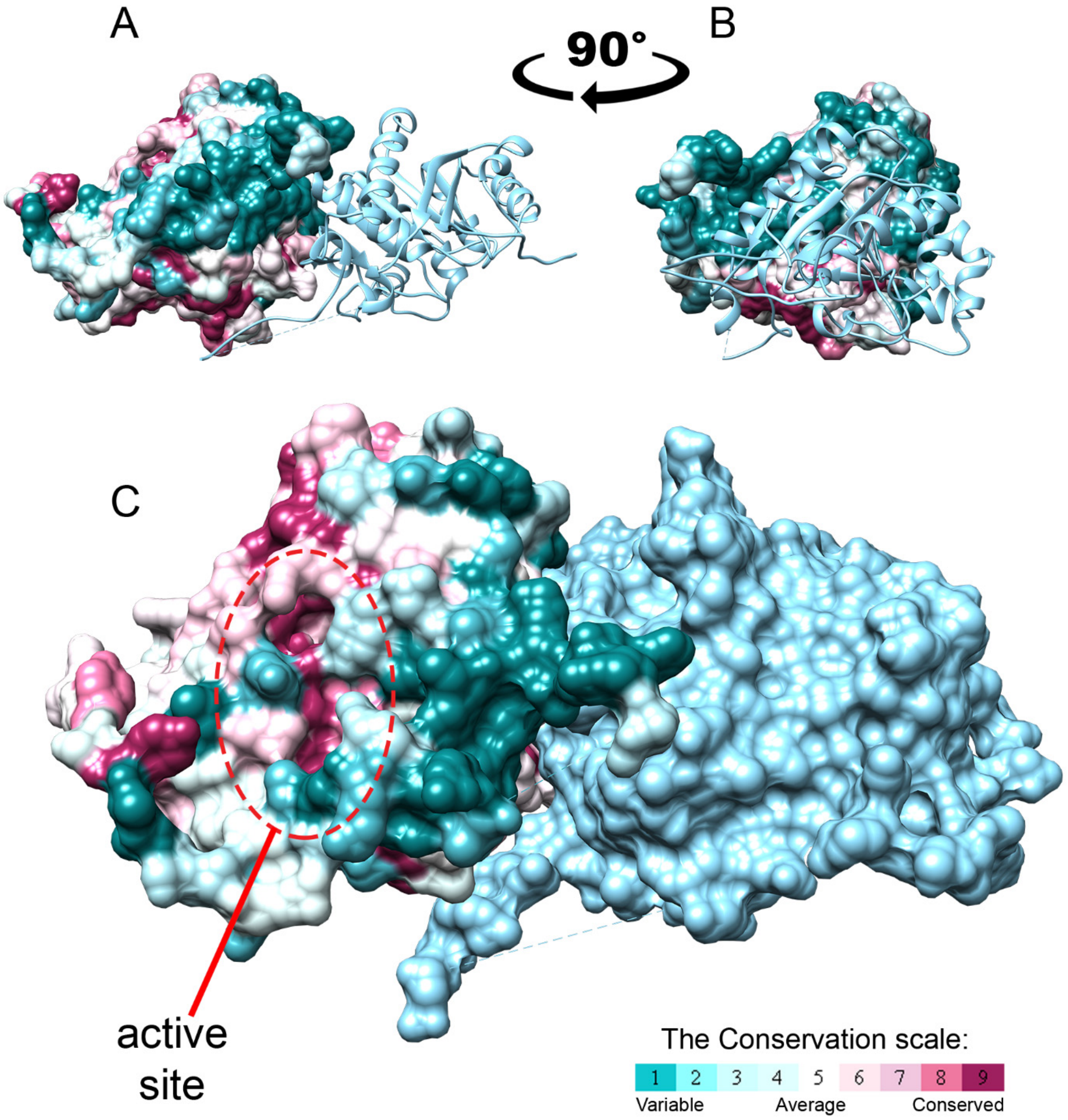

The Etk kinase domain forms a dimer in the crystal structure (PDB id 3CIO). One monomer is colored light blue and the other is colored by evolutionary conservation scores calculated using the ConSurf webserver [14–16]; a variable-to-conserved colored scale is indicated. ADP, present in the crystal structure, is shown in sticks format and colored by atom-type. (A-B) Two views (representing ~90° rotation along the vertical direction) of the Etk dimer with one monomer displayed as ribbons and the other as solvent-accessible surface representation. (C) The two monomers are showed using a solvent-accessible surface representation.
